# BK virus infection and outcome following kidney transplantation in childhood

**DOI:** 10.1038/s41598-021-82160-0

**Published:** 2021-01-28

**Authors:** James McCaffrey, Vijesh J. Bhute, Mohan Shenoy

**Affiliations:** 1 Department of Histopathology, Cambridge University Hospitals NHS Foundation Trust, Addenbrooke’s Hospital, Cambridge, UK; 2 Department of Chemical Engineering, Imperial College London, South Kensington Campus, London, SW7 2AZ UK; 3 Department of Paediatric Nephrology, Royal Manchester Children’s Hospital, Manchester University Hospitals NHS Foundation Trust, Manchester Academic Health Science Centre, Manchester, UK

**Keywords:** Paediatric kidney disease, Infection, Kidney, Kidney diseases, Renal replacement therapy, Viral infection, Infectious diseases, Kidney diseases

## Abstract

BK virus associated nephropathy (BKN) is an important cause of kidney allograft failure. In a cohort of paediatric kidney transplant recipients, we aimed to understand the incidence and clinical outcome associated with BKN, as well as identify risk factors for BKN and BK viraemia development. We retrospectively analysed all patients who received a kidney transplant and received follow up care in our centre between 2009–2019. Among 106 patients included in the study (mean follow up 4.5 years), 32/106 (30.2%) patients experienced BK viraemia. The incidence of BKN was 7/106 (6.6%). The median time of BK viraemia development post-transplant was 279.5 days compared to 90.0 days for BKN. Development of BKN was associated with younger age at transplantation (*p* = 0.013). Development of BK viraemia was associated with negative recipient serology for cytomegalovirus (CMV) at time of transplantation (*p* = 0.012) and a higher net level of immunosuppression (*p* = 0.039). There was no difference in graft function at latest follow up between those who experienced BKN and those without BKN. This study demonstrates that BK virus infection is associated with younger age at transplantation, CMV negative recipient serostatus and higher levels of immunosuppression. Judicious monitoring of BK viraemia in paediatric transplant recipients, coupled with timely clinical intervention can result in similar long-term outcomes for BKN patients compared to controls.

## Introduction

BK virus (BKPyV) is a member of the *Polyomaviridae* family of double-stranded DNA viruses^1^. Primary BKPyV infection is mainly asymptomatic and occurs predominantly before adolescence, with an IgG seroprevalence > 90% in 5–9 year old healthy children. Seroprevalence rates in later life demonstrate an age-dependent decline with 68% of 60–69 year olds showing IgG positivity^2^. Peripheral blood mononuclear cells disseminate BKPyV to the urinary tract where the virus establishes a persistent non-replicative latent phase in renal tubular epithelial cells and urothelium^3,4^. Periodic BKPyV reactivation occurs, with asymptomatic urinary shedding seen in 7% of healthy adults^5^.

In kidney allograft recipients, active replication of BKPyV can lead to BKPyV-associated nephropathy (BKN) and subsequent graft dysfunction and premature loss^6^. The emergence of BKN in the last decade of the twentieth century coincided with the introduction of potent immunosuppressive agents such as tacrolimus and mycophenolate mofetil (MMF), leading to the proposal that a higher level of immunosuppression is a risk factor for the development of BKN^7,8^. BKPyV viraemia precedes the onset of BKN, and polymerase-chain-reaction (PCR) assays of plasma are a specific and sensitive method to detect early nephropathy^9^. Before recognition of the necessity to identify and treat BKN in a timely manner, graft loss rates as high as 67% were documented in the adult population^10^.

Anti-viral agents have demonstrated minimal or no efficacy in clearing BKPyV^11–13^, and treatment involves reducing immunosuppressive therapy to restore the capability of the host immune system to control BKPyV replication and prevent progression to BKN^14^. Levels of BKPyV-specific IgG antibodies and T-cells increase following a reduction in immunosuppression and peak at the time of BKPyV viraemia resolution^15^.

In the paediatric kidney transplant population, rates of BKPyV viraemia range from 18–37% and BKN is identified in 0–16% of patients^8,15–23^. In a case series of 32 patients under the age of 20 years with BKN, 3 (9%) of allografts were lost^6^. Reported risk factors for the development of BKN in the paediatric population include allograft recipient BKPyV seronegativity at time of transplantation^20,23^, zero human leukocyte antigens (HLA) -A and -DR mismatches between transplant donor and recipient^22^, increased levels of immunosuppression,^8,21^ younger age of recipient at transplantation^21^, and a tacrolimus- compared to ciclosporin- based immunosuppression regimen^21^. A recent study in adult kidney transplant recipients suggested prophylactic use of the anti-cytomegalovirus (CMV) agent valganciclovir may be associated with an increased risk of BKN^24^.

In this single-centre study, we report our experience of BKN in a paediatric transplant recipient population over a 10-year period, with patients predominantly receiving a steroid-sparing immunosuppressive regimen^25^. Specific aims of the study were to: (1) evaluate rates of BKPyV viraemia and BVN in this population; (2) understand the morbidity associated with BKPyV viraemia and BKN; and (3) identify risk factors for the development of BKPyV viraemia and BKN.

## Results

### Study population

A total of 106 patients met the requirements for inclusion in the study, with a mean follow up time of 54.3 months. There were 7 graft failures (mean time in months post-transplant: 44.0 months, range 18–70 months) and 2 deaths (see Supplementary Table S1). There were no graft losses or deaths in BKN_Hi/B+_ patients (Table 1). 99/106 patients received the steroid-sparing TWIST immunosuppressive regimen. 7/106 patients received a non-TWIST immunosuppression regimen (reasons including enrolment in research trials and transplant occurring before TWIST became standard local protocol). Characteristics for the whole cohort are provided in Supplementary Tables S2, S3 and S4.

**Table 1:** Univariate analysis of patient cohort characteristics.

Characteristic	No-BKV (n = 74)		BKN_Low_ (n = 25)		BKN_Hi/B+_ (n = 7)		*p* value
	Proportion (%)	Mean	Proportion (%)	Mean	Proportion (%)	Mean	
Female	24/74 (32.4%)		9/25 (36.0%)		2/7 (28.6%)		0.939
Male	50/74 (67.6%)		16/25 (64.0%)		5/7 (71.4%)		
Age at transplant (years)		11.2		10.2		6.2	0.0369
Ethnicity: White British^+^	45/68 (66.2%)		17/25 (68.0%)		5/7 (71.4%)		1.000
Living transplant donor	43/74 (58.1%)		18/25 (72.0%)		6/7 (85.7%)		0.228
Cold ischaemia time (minutes)		345.2		235.6		143.9	0.218
Steroid-free regimen at latest follow up	44/74 (59.5%)		11/25 (44.0%)		4/7 (57.1%)		0.448
MMF part of regimen at latest follow up	55/74 (74.3%)		16/25 (64.0%)		5/7 (71.4%)		0.543
Exposure to anti-CMV therapy	24/74 (32.4)		10/25 (40.0%)		3/7 (42.9%)		0.735
BPAR	21/74 (28.4)		6/25 (24.0%)		2/7 (28.6%)		0.933
Graft failure	7/74 (9.5%)		1/25 (4.0%)		0/7 (0.0%)		0.802
Death	2/74 (2.7%)		0/25 (0.0%)		0/7 (0.0%)		1.000
Length of follow up (months)	55.8		48.0		60.1		0.453

^+^Data not available for six patients. BPAR = biopsy proven acute rejection.

### Incidence of BKN

There were 41 episodes of BK viraemia in 32/106 patients (30.2%). 2/106 patients (1.9%) experienced an episode of BKN_B+_, and a further 5/106 (4.7%) patients experienced an episode of BKN_Hi_. Therefore, the incidence of BKN_Hi/B+_ was 7/106 (6.6%). 25/106 patients (23.6%) experienced an episode of BKN_Low_ without an episode of BKN_Hi/B+_ at any point post-transplantation.

### Timing of episodes

**Figure 1:**
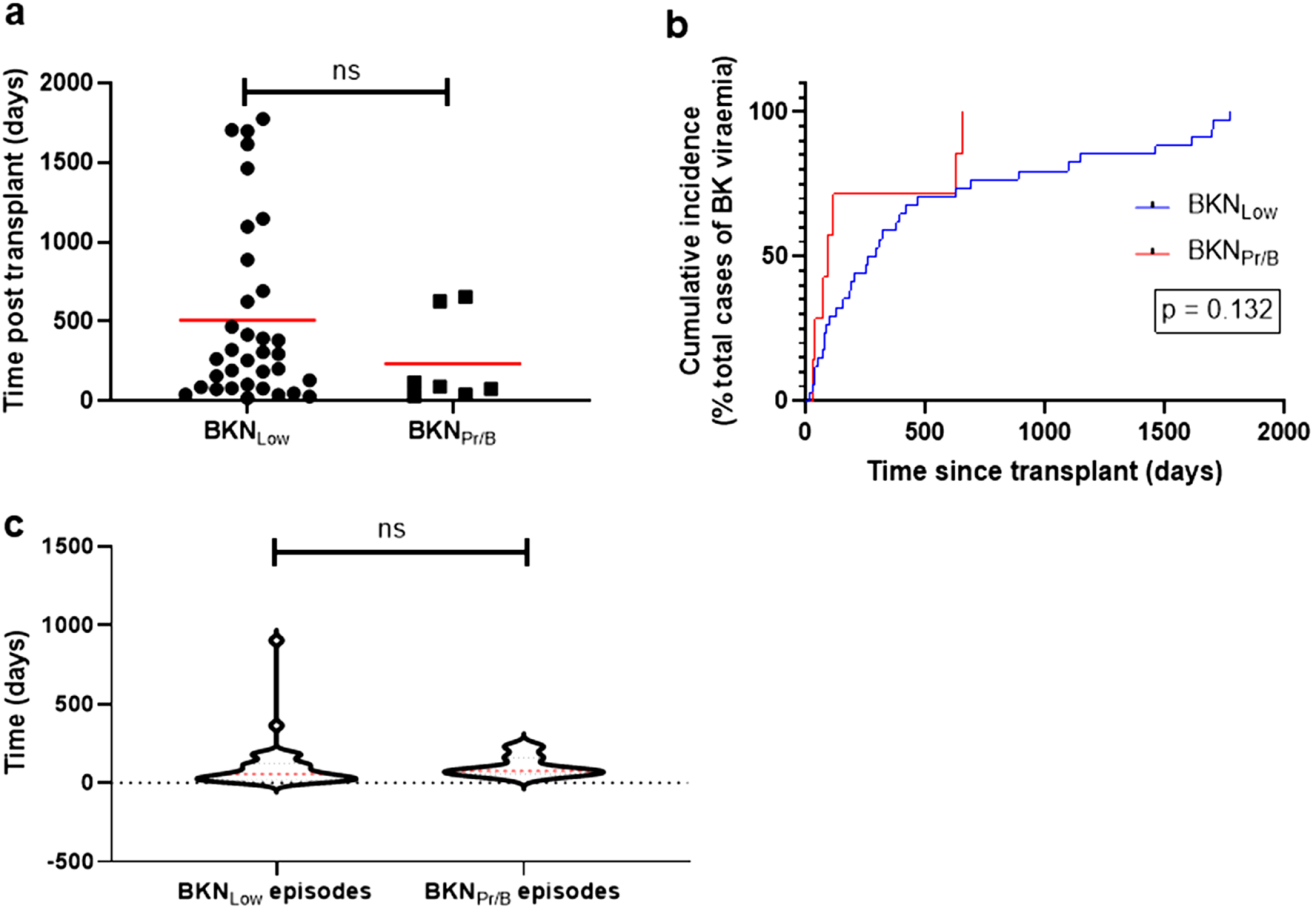
Characteristics of BKyPV viraemia episodes. (**a**) Time of onset of viraemia episodes post-transplant (*p* = 0.199, Mann–Whitney U test). Red line indicates mean value for each group. (**b**) Cumulative risk of developing BKPyV viraemia over time (curves compared using log-rank / Mantel-Cox test). (**c**) Violin plot comparing length of viraemia episodes between groups (*p* = 0.265, Mann–Whitney U test). Quartiles are plotted with median value in red. ns = not significant.

The mean (median) time of viraemia onset post-transplant in the 34 episodes of BKN_Low_ was 508.4 (279.5) days compared to 234.3 (90.0) days for BKN_Hi/B+_ (*p* = 0.199) (Fig. 1a and Supplementary Table S5). The range of time of onset of viraemia post-transplant was 19–1775 days for BKN_Low_ episodes and 33–656 days for BKN_Hi/B+_. 23.5% of BKN_Low_ episodes occurred more than 2 years post transplant. In contrast, all episodes of BKN_Hi/B+_ occurred within the first 2 years following transplantation.

**Figure 2:**
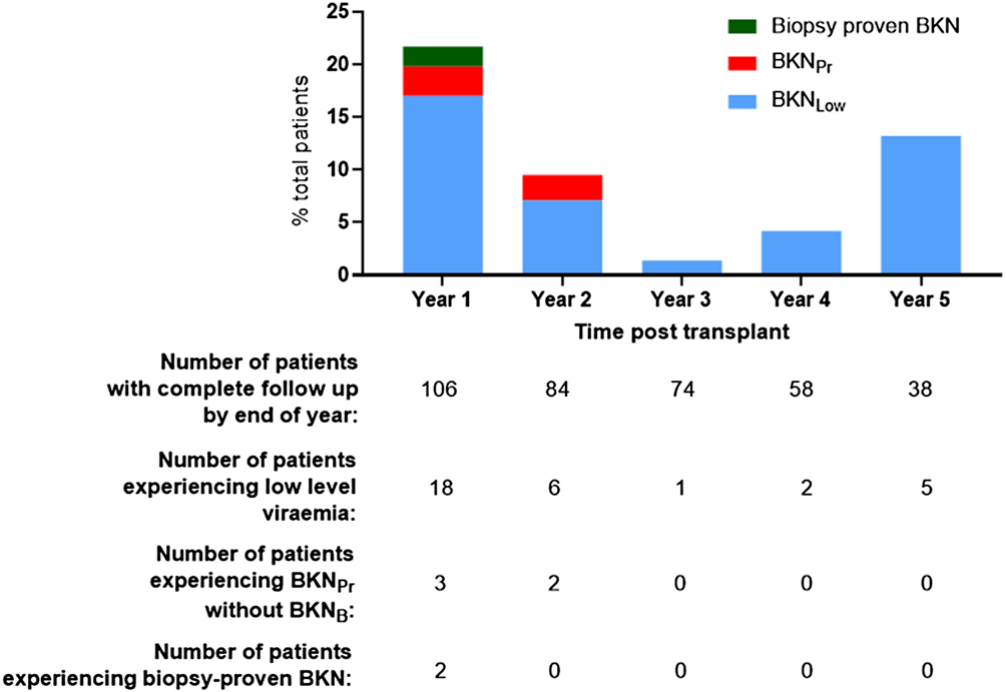
Incidence of BKN_Low_, BKN_B+_ and BKN_Hi_ in years 1–5 post transplantation.

12/34 (35.3%) of total BKN_Low_ episodes occurred in the 6 months immediately post-transplant compared to 5/7 (71.4%) episodes of BKN_Hi/B+_. A plot of the Kaplan–Meier estimator demonstrated no significant difference between the timing of onset of BKN_Low_ and BKN_Hi/B+_ viraemia (Fig. 1b). An overview of the temporal distribution of BK viraemia episodes is provided in Fig. 2.

No difference between length of viraemia per episode was noted between BKV_All_ subgroups (mean 105.8 days for BKN_Low_ compared to 104.0 days for BKN_Hi/B+_, *p* = 0.265) (Fig. 1c and Supplementary Table S6).

### Analysis of risk factors

Univariate analysis was performed to assess potential risk factors for the development of BKPyV viraemia and BKN. Age at transplant was significantly different between the 3 groups (*p* = 0.0369) (Table 1). Bonferroni’s post-test was used to compare groups and there was a significant difference in age at transplantation between no-BKV patients (mean 11.2 years) compared to BKN_Hi/B+_ patients (mean 6.2 years) (*p* = 0.013, adjusted critical value α_Bonferroni_ = 0.0167 for three group comparison). There was no difference between age at transplantation between no-BKV patients and BKN_Low_ patients (mean 10.2 years) (*p* = 0.363).

CMV serology status of allograft recipients was also significantly different between the groups (*p* = 0.012) (Table 2). BKN_Low_ patients were less likely to have recorded positive CMV serology at time of transplantation (2/25, 8.0%) than no-BKV (27/74. 36.5%) and BKN_Hi/B+_ (3/7, 42.9%) patients. There was no difference in exposure to anti-CMV therapy between patient sub-groups (Table 1 and Supplementary Table S7).

**Table 2:** Univariate analysis of CMV serostatus of transplant donor and recipient.

		No BK Proportion (%)	BKN_Low_ Proportion (%)	BKN_Hi/B+_ Proportion (%)	*p* value
CMV status donor	+	29/74 (39.2%)	11/25 (44.0%)	3/7 (42.9%)	0.944
	−	45/74 (60.8%)	14/25 (56.0%)	4/7 (57.1%)	
CMV status recipient	+	27/74 (36.5%)	2/25 (8.0%)	3/7 (42.9%)	0.012
	−	47/74 (63.5%)	23/25 (92.0%)	4/7 (57.1%)	

No differences between patient subgroups were identified for the following characteristics: sex, cold ischaemia time, ethnic background, frequency of BPAR, use of steroids/MMF, source of graft donor (living/cadaveric) and HLA mismatch status (Table 1 and Supplementary Table S8).

Multinomial logistic analysis confirmed the univariate analysis findings of CMV recipient serostatus and age at time of transplant being independently predictive of BKN and BKPyV viraemia. Additionally, age and CMV serostatus in the same model are predictive of BKN/ BKPyV viraemia (*p* = 0.002) (Table 3).

**Table 3:** Multinomial Logistic Regression Analysis.

Factor	*p* value of reduced model^a^	*p* value of final model	BKN_Low_ status^d^			BKN_Hi/B+_ status^d^		
			OR^b^	95% CI^c^	*p* value	OR^b^	95% CI^c^	*p* value
Age at transplant	0.027	0.002	0.975	0.889 to 1.070	0.594	0.785	0.636 to 0.968	0.024
CMV serostatus of recipient	0.009		0.157	0.034 to 0.723	0.018	1.869	0.350 to 9.968	0.464

^a^Log Likelihood tests ; ^b^OR: odds ratio; ^c^CI: confidence interval for odds ratio; ^d^Reference category is No-BKV.

### Immunosuppression

**Figure 3:**
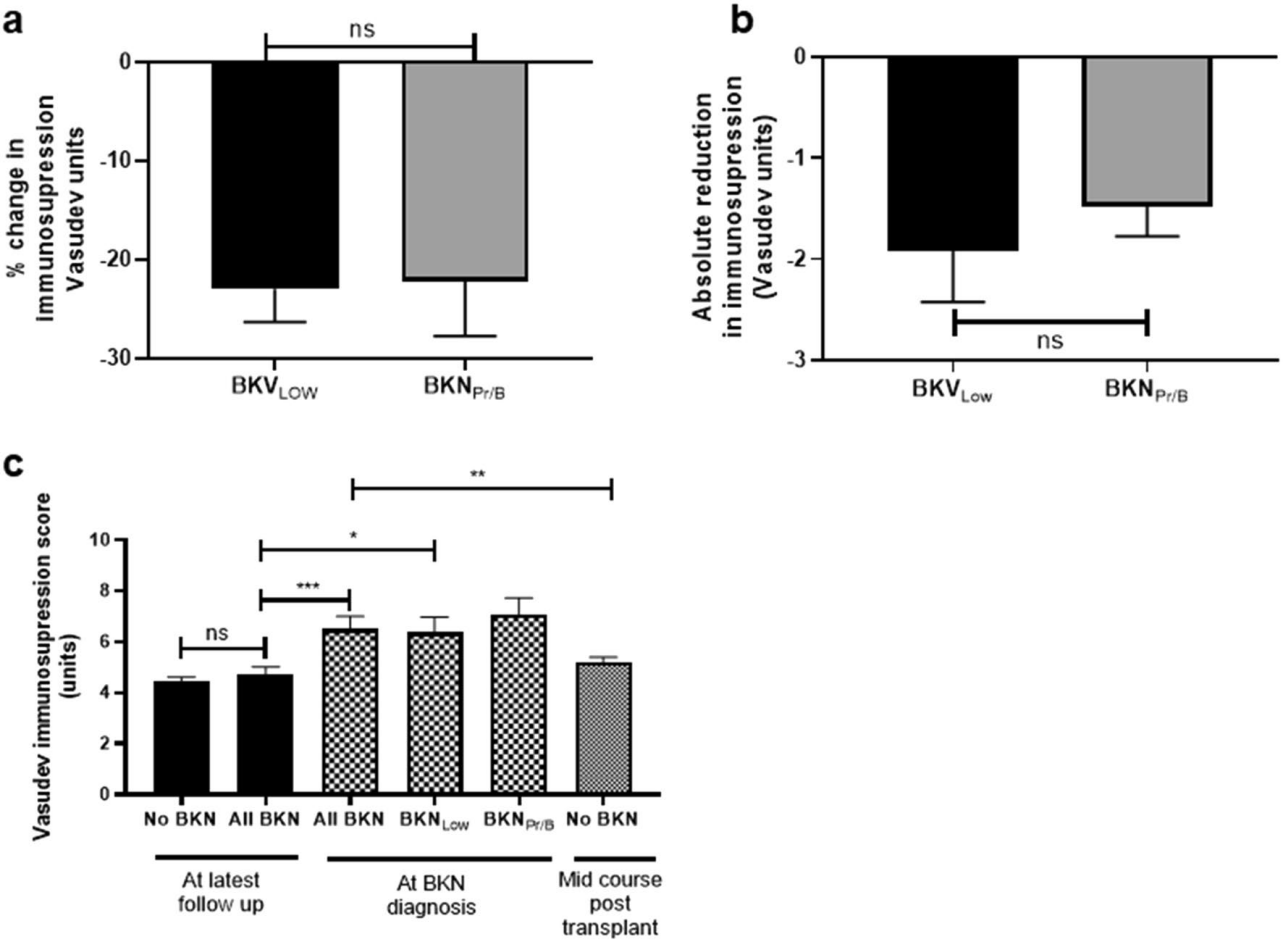
Change in net immunosuppression over time. (**a**) Percentage change in paediatric immunosuppression Vasudev score during episodes of BKPyV viraemia (*p* = 0.588, Mann–Whitney U test). (**b**) Absolute change in paediatric immunosuppression Vasudev score during episodes of BKPyV viraemia (*p* > 0.999, Mann–Whitney U test). **c** Mean paediatric Vasudev immunosuppression score at latest follow up, at BKPyV viraemia diagnosis and at 462 days post-transplantation in no-BKV group. Groups compared using one way ANOVA followed by Tukey’s test. ns = not significant (*p* = 0.996), ***p = 0.0115, **p = 0.0390, *p = 0.0391.

All (7/7) episodes of BKN_Hi/B+_ elicited a reduction in immunosuppression by the treating clinician compared to 13/34 (38.2%) of episodes of BKN_Low_ (Supplementary Table S9). However, when the decision to reduce immunosuppression was made, there was no significant difference between the size of reduction between BKN_Low_ (mean reduction in paediatric Vasudev score − 23.0%) and BKN_Hi/B+_ (mean reduction in paediatric Vasudev score -22.2%) (Fig. 3a,b).

To understand how the total level of immunosuppression varied for patients across their post-transplant clinical course, a paediatric Vasudev score was calculated for all patients at latest follow up and, for relevant patients, at time of BKPyV viraemia onset. Additionally we quantified the level of immunosuppression for no-BKV patients at the mean time BKV_All_ patients developed viraemia post-transplant (462 days). This allowed us to analyse no-BKV and BKV_All_ patients at comparable points in their clinical course.

There was no significant difference in paediatric Vasudev score at latest follow up between no-BKV patients (mean 4.4 units) compared to BKV_All_ patients (mean 4.7 units) (Fig. 3c). However, the level of immunosuppression was significantly higher for BKV_All_ patients at time of BKPyV viraemia diagnosis (mean 6.5 units) compared to latest follow-up (*p* = 0.012). Furthermore, immunosuppression level in no-BKV patients at 462 days post-transplantation (mean 5.2 units) was significantly lower than for BKV_All_ patients at BKPyV viraemia diagnosis (*p* = 0.0390). This shows that patients who developed BKPyV viraemia were exposed to a higher level of immunosuppression at time of diagnosis than patients without BKPyV viraemia.

To understand if increases in immunosuppression following an episode of acute rejection may explain the higher paediatric Vasudev score recorded for BKV_All_ patients, we examined whether BKPyV viraemia or acute rejection occurred first for each relevant patient. There were 8 episodes of BPAR among BKV_All_ patients: in 4/8 episodes rejection occurred before BKPyV viraemia and in 4/8 episodes BKPyV viraemia occurred before rejection. 2/2 BKN_B+_ patients experienced an episode of acute rejection: in both instances BKN occurred before acute rejection.

### Effect on kidney function

**Figure 4:**
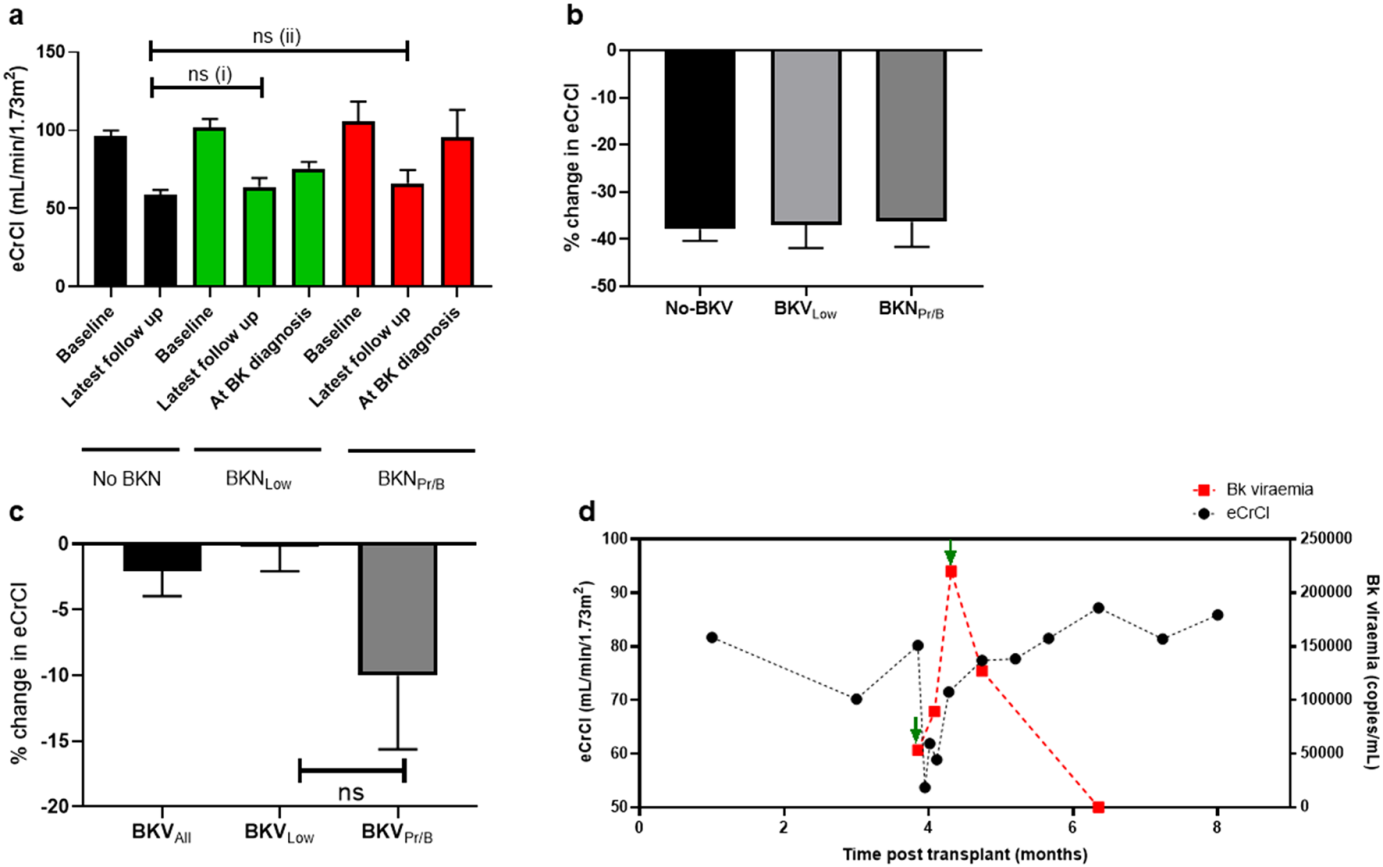
Effect of BKPyV viraemia on graft function. (**a**) Estimated creatinine clearance (eCrCl) at baseline, latest follow up and at BKPyV viraemia diagnosis. Black bars = ‘No-BKV’, green bars = ‘BKN_Low_’, red bars = ‘BKN_Hi/B+_’. [ns (1) *p* = 0.996, ns (2) p = 0.998, one way ANOVA followed by Tukey’s test]. (**b**) Percentage change in eCrCl at latest follow up compared to baseline. (**c**) Percentage change in lowest eCrCl recorded during BKPyV viraemia compared to eCrCl at BKPyV viraemia diagnosis (ns *p* = 0.109, Mann–Whitney U test). (**d**) Representative plot showing plasma viral load and eCrCl of a patient with BKN_Hi_ over time. Green arrows indicate points at which immunosuppressive therapy was reduced.

There was no significant difference in eCrCl between patient sub-groups at latest follow up or significant difference in the eCrCl percentage change from baseline to latest follow up between patient sub-groups (Fig. 4a,b).

To analyse changes in kidney function during periods of viraemia, the eCrCl at BKPyV viraemia diagnosis was compared to the lowest eCrCl during the period of viraemia for each patient and a percentage change was calculated. BKN_Low_ patients showed a mean eCrCl change of − 0.2%, while BKN_Hi/B+_ patients showed a mean eCrCl change of − 10.0% (Fig. 4c). However, there was no significant difference between the eCrCl at BKPyV viraemia diagnosis and the average eCrCl reading during viraemia for either BKN_Low_ or BKN_Hi/B+_ patients (Supplementary Figure S1). A representative plot of plasma viral load and eCrCl of a patient with BKN_Hi_ is shown in Fig. 4d.

## Discussion

The salient findings of this study involving 106 paediatric kidney transplant recipients are as follows: (1) 2/106 individuals experienced BKN_B+_ and 5/106 patients developed BKN_Hi_, giving an overall BKN incidence of 7/106 (6.6%); (2) 30.2% patients experienced BKPyV viraemia; (3) the incidences of BKN_Low_, BKN_B+_ and BKN_Hi_ were highest during the first year post-transplant; (4) 23.5% of BKN_Low_ episodes occurred after 2 years post-transplantation; (5) there was no significant difference in long-term graft function between patients experiencing BKPyV viraemia/BKN and no-BK controls; (6) younger age at transplantation, CMV negative serology of the allograft recipient at time of transplantation, and a higher level of immunosuppression were associated with an increased risk of BKN/ BKPyV viraemia development.

Our incidence of BKN_B+_ (1.9%) is slightly lower than rates of 3.5–4.5% recorded in previous studies^20–22^. We found similar levels of BKPyV viraemia to a recent European mulitcentre study (30.2% vs. 36.7%), but lower level of BKN_Hi_ (4.7% vs. 15.8%)^21^. There were no graft losses from BKN_Hi/B+_ patients in our cohort, whereas graft failure rates of 7.1–24.0% have previously been recorded in American and European data^21,22^.

There was no difference in eCrCl at final follow up between patients experiencing BKPyV viraemia or BKN and patients without BKPyV viraemia / BKN. Our graft outcome findings are similar to a prospective study of 62 paediatric transplants which reported no difference in SCr between patients who developed BKPyV viraemia and those who did not (36 months follow up post-transplantation)^15^. Data from adult transplant recipients indicated that immunosuppression can safely be reduced in patients with BKPyV viraemia to prevent progression to BKN, without a concomitant increase in the incidence of acute rejection^14^. However, some paediatric data suggested an increased risk of BKPyV viraemia in patients who had experienced an episode of acute rejection^15^. In the current study, we found no difference in rates of BPAR among patient sub-groups, and in the 8 episodes of BPAR occurring in BKV_All_ patients, only half preceded BKPyV viraemia development. These observations suggest that increases in immunosuppression due to rejection are unlikely to be a major contributor to BKPyV viraemia development in our cohort.

Rates of BKPyV viraemia in the current study exceed those found in the adult transplant population (11.5–12.8%)^14,26^, and suggests children may have an increased risk of developing BKPyV-associated pathology. Indeed, we identified younger age at transplantation as a risk factor for BKN development. As BKPyV seropositivity increases from 6% in the 1–4 year age range to 91% in the 5–9 year age range^26^, it is likely that a proportion of the BKN episodes in our cohort among the younger children represented primary BKPyV infection. No definitive evidence exists demonstrating that primary BKPyV replication in seronegative individuals results in a poorer clinical outcome compared to secondary BKPyV replication in seropositive individuals^6^. However, our finding is in agreement with other studies^21^, and suggests younger transplant recipients may benefit from increased frequency of monitoring and rapid instigation of treatment to prevent uncontrolled viral replication.

We found that CMV seronegative individuals were more likely to develop BKPyV viraemia than CMV seropositive patients. Following a recent report linking prophylactic valganciclovir use with an increased risk of BKN^24^, we investigated whether anti-CMV therapy (routinely prescribed in CMV negative individuals) was associated with an increased risk of BKPyV replication in our cohort. However, we found no evidence to support this (Table 1 and Supplementary Table S7). CMV seropositivity rapidly increases with age during childhood in a similar manner to rates of BKPyV seropositivity^27^. It is therefore possible that seronegative CMV individuals are more likely to also be BKPyV seronegative, and therefore at heightened risk of uncontrolled viral replication during primary BKPyV infection. However, due to the retrospective nature of the study, this conjecture cannot be confirmed.

Our data supports previous observations that a higher level of immunosuppression is a risk factor for BKPyV reactivation. A tacrolimus/MMF-based immunosuppression regimen has been shown to double the risk of BKPyV viraemia compared to a ciclosporin /MMF-based regimen^21^. However, rates of BKPyV viraemia in our cohort (predominantly tac/MMF-based regimen) are comparable to those treated with alternative regimens.

Limitations of the current study include the retrospective approach and relatively small number of subjects in comparison to multi-centre investigations. In particular, the low numbers of patients experiencing BKN in this study reduces the confidence with which definitive conclusions can be drawn from these data. Additionally, a prospective approach would have allowed collection of biological samples at various time points throughout a patient’s clinical course to interrogate BKPyV serostatus at the time of transplantation and understand the effect seronegativity has on clinical outcome.

Overall, our results demonstrate that control of BKPyV replication in paediatric transplant recipients can be attained through judicious surveillance and timely reduction in immunosuppression, without an increased risk of acute rejection. This approach results in comparable graft function and survival outcomes between patients developing BKN and those without evidence of viraemia. The findings that younger age at transplantation significantly increases a patient’s risk of developing BKN, and almost a quarter of BKPyV viraemia episodes occur later than 2 years post-transplant has implications for the development of BKN surveillance strategies.

## Methods

### Study design

This is a retrospective cohort analysis of patients receiving a kidney transplant at the Royal Manchester Children’s Hospital (RMCH) (UK). Case notes were reviewed from June 2009 to January 2019 and patients who met the following requirements were included in the study: (1) Aged < 19 years at time of transplantation; (2) a minimum of 12 months follow-up data; and (3) transplant performed at RMCH. For patients who received more than one transplant, the first transplant was evaluated.

Clinical and serological data were collected at time of transplant and at months 1, 3, 6, 9, 12 months post-transplantation and at 6 monthly intervals thereafter. All plasma BKPyV quantification and histology results were recorded. Immunosuppression regimens for each patient were documented at latest follow up, and during BKPyV viraemia episodes for relevant patients. Contemporaneous patient height and weight results were recorded for each serum creatinine (SCr) reading, to allow the estimated creatinine clearance (eCrCl) to be calculated using the standard Schwartz formula (k = 40, as per local protocol)^28^. All SCr readings during periods of BKPyV viraemia were recorded. The study protocol was reviewed by the Clinical Trial Management Department (Manchester University NHS Foundation Trust) who waived ethical approval for this study involving the retrospective collection of anonymised data. They concluded that informed consent was not required from study participants. The study was performed in accordance with Manchester University NHS Foundation Trust guidelines. No patient identifiable data was recorded.

### BKPyV surveillance and immunosuppression regimen

Plasma BKPyV monitoring is performed in all kidney transplant recipients every month during the first year post-transplantation and on an annual basis thereafter using a TaqMan PCR-based approach. Monitoring is performed more frequently at the treating clinician’s discretion.

The standard local immunosuppression approach is the steroid-sparing (TWIST) protocol^25^. Briefly, this involves induction therapy with the interleukin 2 receptor monoclonal antibody antagonist, basiliximab, on day 0 and day 4 post transplantation, coupled with a rapidly weaning course of prednisolone which is discontinued on day 5. Long-term immunosuppression is provided by tacrolimus and MMF. The net immunosuppressive load at different time points for each patient was quantified using the paediatric Vasudev score^29^. This score provides an estimation of the overall immunosuppressive load by integrating the immunosuppressant types, doses and body surface area of each patient. For example, a patient administered 1.2 mg/m^2^ per day of tacrolimus (equating to a paediatric Vasudev score of 1 unit) and 580 mg/m^2^ per day of MMF (equating to a paediatric Vasudev score of 2 units), would have a total paediatric Vasudev score of 3 units.

### Definitions

Patients with no recorded positive BKPyV plasma result or histological evidence of BKN were designated as ‘no-BKV.’ Patients with any recordable level of BKPyV viraemia at any time during study follow-up were designated as ‘BKV_All_.’ The BKV_All_ group was subdivided into three groups: (1) those with biopsy-proven BKN (‘BKN_B+_’); (2) those with presumptive BKN (‘BKN_Hi_’) without histological evidence of BKN. BKN_Hi_ is defined as a sustained (> 3 week) high-level viraemia (> 10^4^ copies/mL), as per international recommendations^21,30^; and (3) patients with BKPyV viraemia but not meeting the requirements for either BKN_B+_ or BKN_Hi_ (‘BKN_Hi/B+_’), were designated as having low-level viraemia (‘BKN_Low_’).

The length of viraemia for each episode was defined as the length of time from the first positive BKPyV plasma viraemia result until plasma levels were again undetectable. Baseline eCrCl was defined as the highest eCrCl recorded during the first year following transplantation. Graft failure was defined as the requirement to return to dialysis after the first week post-transplantation.

### Statistical analysis

Univariate analysis of risk factors was performed using Fisher’s exact test and chi-squared test for correlation (for categorical variables) and one-way analysis of variance (ANOVA) with Bonferroni’s post-hoc test (for quantitative variables). Analysis of clinical outcomes was performed using the non-parametric Mann–Whitney U test or one-way ANOVA with Tukey’s test, as appropriate.

### Multinomial analysis

A multinomial logistic regression analysis was used to predict the BKN development. The following variables were considered for the model: gender, ethnicity, age at transplant, cold ischaemia time, biopsy-proven acute rejection (BPAR), source of allograft (cadaveric/living), use of steroid-free regimen at latest follow-up, use of MMF at latest follow up, exposure to anti-CMV therapy, HLA mismatch, recipient CMV status, donor CMV status. The analysis was performed in IBM SPSS Statistics Version 26. A parsimonious model was developed by using forward selection method. The final model consisted of one factor: CMV serostatus of recipient and one covariate: age at transplant.

## Supplementary Information

Supplementary Information.
