# Supplementary material for: BK virus infection and outcome following kidney transplantation in childhood
Source: Sci Rep. 2021 Jan 28;11:2468. doi: 10.1038/s41598-021-82160-0 (PMC7844021; doi:10.1038/s41598-021-82160-0)
Supplement: Supplementary file 1 — Supplementary Information. [file 41598_2021_82160_MOESM1_ESM.docx]

**Supplementary information**

**BK Virus infection and outcome following kidney transplantation in childhood**

James McCaffrey^1,*^, Vijesh J. Bhute^2^, Mohan Shenoy^3^

*1. Department of Histopathology, Cambridge University Hospitals NHS Foundation Trust, Addenbrooke's Hospital, Cambridge, UK. 2. Department of Chemical Engineering, Imperial College London, South Kensington Campus, London SW7 2AZ, U.K. 3. Department of Paediatric Nephrology, Royal Manchester Children's Hospital, Manchester University Hospitals NHS Foundation Trust, Manchester Academic Health Science Centre, Manchester, UK.*

*Corresponding author:

Dr James McCaffrey

Cambridge University Hospitals NHS Foundation Trust, Addenbrooke's Hospital, Cambridge, UK

Email: jm2199@cam.ac.uk

**Supplementary Table S1** Details of two patient deaths in cohort

| **Patient** | **Cause of renal failure** | **Time from transplant to death (months)** | **Age at death** | **BK viraemia category** | **Cause of death** |
| --- | --- | --- | --- | --- | --- |
| 1 | Methylmalonic acidaemia | 14 | 15 years, 8 months | No-BK | Acute pancreatitis, encephalopathy, pneumonia |
| 2 | Dysplasia | 49 | 12 years, 11 months | No-BK | Acute lymphoblastic leukaemia |

**Supplementary Table S2**  Characteristics of whole patient cohort

|  | **Proportion (%)** | **Mean** | **Median** | **Range** |
| --- | --- | --- | --- | --- |
| Female | 35/106 (33.0%) |  |  |  |
| Male | 71/106 (67.0%) |  |  |  |
| Age at transplant (years) |  | 10.7 | 10.5 | 2.5 – 18.3 |
| Length of follow-up (months) |  | 54.3 | 49 | 12 – 137 |
| Cold ischaemia time (mins) |  | 304.4 | 65 | 29 – 1218 |
| Living donor transplant | 67/106 (63.2%) |  |  |  |
| Deceased donor transplant | 39/106 (36.8%) |  |  |  |
| Steroids in immunosuppression regimen at latest follow up | 47/106 (44.3%) |  |  |  |
| MMF in immunosuppression regimen at latest follow up | 76/106 (71.7%) |  |  |  |
| BPAR | 29 / 106 (27.3%) |  |  |  |
| Graft failure | 7/106 (6.6%) |  |  |  |
| Death | 2/106 (1.9%) |  |  |  |

BPAR=biopsy proven acute rejection

**Supplementary Table S3** Causes for end stage kidney disease in patient cohort

| **Cause of renal failure** | **Proportion (%)** |
| --- | --- |
| Dysplasia alone | 29/106 (27.4%) |
| Steroid resistant nephrotic syndrome, not otherwise specified | 11/106 (10.4%) |
| PUV/ dysplasia | 16/106 (15.1%) |
| Ciliopathy | 10/106 (9.4%) |
| Cystinosis | 6/106 (5.7%) |
| Unknown | 6/106 (5.7%) |
| Congenital nephrotic syndrome | 3/106 (2.8%) |
| PCKD | 3/106(2.8%) |
| MMA | 3/106 (2.8%) |
| Reflux nephropathy | 3/106(2.8%) |
| VACTERL syndrome | 2/106 (1.9%) |
| VUR and dysplasia | 1/106(0.9%) |
| Alport syndrome | 1/106 (0.9%) |
| Denys Drash syndrome | 1/106(0.9%) |
| Neonatal bilateral renal vein thrombosis | 1/106(0.9%) |
| pANCA positive vasculitis | 1/106(0.9%) |
| Post-infectious glomerulonephritis | 1/106(0.9%) |
| Prune belly syndrome | 1/106(0.9%) |
| Brachio oto renal syndrome | 1/106(0.9%) |
| C3 glomerulonephritis | 1/106(0.9%) |
| Familial cystic nephropathy | 1/106(0.9%) |
| Frasier syndrome | 1/106(0.9%) |
| HSP nephritis | 1/106(0.9%) |
| HSP/IgA nephropathy | 1/106(0.9%) |
| HUS | 1/106(0.9%) |

PCKD= polycystic kidney disease, MMA= methylmalonic acidaemia, VUR= vesicoureteral reflux, pANCA= perinuclear anti-neutrophil cytoplasmic antibodies, HSP= Henoch-Schönlein purpura, IgA= immunoglobulin A, HUS= Haemolytic uraemic syndrome, PUV= posterior urethral valves

**Supplementary Table S4** Ethnic background of patient cohort

| **Ethnicity** | **Proportion (%)** |
| --- | --- |
| White – British | 67/106 (63.2%) |
| Asian / Asian British – Pakistani | 21/106 (19.8%) |
| White - Any other white background | 1/106 (0.9%) |
| Mixed - White & Black Caribbean | 1/106 (0.9%) |
| Mixed - Any other mixed background | 1/106 (0.9%) |
| Asian / Asian British – Indian | 1/106 (0.9%) |
| Asian / Asian British – Bangladeshi | 3/106 (2.8%) |
| Black / Black British – African | 1/106 (0.9%) |
| Any other ethnic group | 1/106 (0.9%) |
| Not recorded | 6/106 (5.7%) |

**Supplementary Table S5** Timing of BK viraemia episodes

|  | **BKV_All_ episodes** | **BKN_Low_ episodes** | **BKN_Hi/B+_ episodes** |
| --- | --- | --- | --- |
| Mean time post transplant (days) | 461.6 | 508.4 | 234.3 |
| Median time post transplant (days) | 256.0 | 279.5 | 90.0 |
| Range (days) | 19.0 – 1775.0 | 19.0 – 1775.0 | 33.0 – 656.0 |
| Proportion of episodes occurring <6months post transplant | 17/41 (41.5%) | 12/34 (35.3%) | 5/7 (71.4%) |
| Proportion of episodes occurring 6 months – 1 year post transplant | 8/41 (19.5%) | 8/34 (23.5%) | 0/7 |
| Proportion of episodes occurring 1-2 years post transplant | 8/41 (19.5%) | 6/34 (17.6%) | 2/7(28.6%) |
| Proportion of episodes occurring > 2years post transplant | 8/41 (19.5%) | 8/34 (23.5%) | 0/7 |

**Supplementary Table S6** Characteristics of BKPyV viraemia episodes

|  | **Proportion (%)** | **Mean** | **Median** | **Range** |
| --- | --- | --- | --- | --- |
| Patients experiencing at least one episode of BK viremia of any level | 32/106 (30.2%) |  |  |  |
| Patients experiencing only low level BK viraemia | 25/106 (23.6%) |  |  |  |
| Patients experiencing at least one episode of BKN_Hi/B+_ | 7/106 (6.6%) |  |  |  |
| Maximum level of viremia per episode (copies/mL): Low-level viremia |  | 2105 | 296.0 | 18.0 – 17105.0 |
| Maximum level of viremia per episode (copies/mL) : BKN_Hi/B+_ |  | 77407 | 77909 | 576.0 – 220078.0 |
| Time to clearance in low level BK viraemia episodes (days) |  | 105.8 | 55.0 | 7.0 -903.0 |
| Time to clearance in BKN_Hi/B+_ (days) |  | 104.0 | 76.0 | 43.0 – 230.0 |
| Creatinine clearance at latest follow up : No-BKV episodes |  | 59.1 | 60.9 | 13.3-188.4 |
| Creatinine clearance at latest follow-up: only low level BK viraemia |  | 64.0 | 56.1 | 10.8-121.4 |
| Creatinine clearance at latest follow up: BKN_Hi/B+_ |  | 66.4 | 62.7 | 34.7-95.9 |

|  | **All patients** | **No-BKV patients** | **BKV_All_ patients** | **BKN_Low_ patients** | **BKN_Hi/B+_ patients** |
| --- | --- | --- | --- | --- | --- |
| No anti-CMV therapy | 69/106 (65.1%) | 50/74 (67.6%) | 19/32 (59.4%) | 15/25 (60.0%) | 4/7 (57.1%) |
| Valganciclovir – treatment dose only | 3/106 (2.8%) | 3/74 (4.1%) | 0 | 0 | 0 |
| Valganciclovir – prophylaxis dose only | 26/106 (24.5%) | 17/74 (22.9%) | 9/32 (28.1%) | 6/25 (24.0%) | 3/7 (42.9%) |
| Valganciclovir – treatment and prophylaxis | 5/106 (4.7%) | 2/74 (2.7%) | 3/32 (9.4%) | 3/25 (12.0%) | 0 |
| IV ganciclovir, Valganciclovir treatment and prophylaxis | 2/106 (1.9%) | 1/74 (1.4%) | 1/32 (3.1%) | 1/25 (4.0%) | 0 |

**Supplementary Table S7** Table detailing exposure to anti-CMV therapy in cohort

sub-groups

**Supplementary Table S8** Univariate analysis of HLA mismatches and BKN status

|  | **No-BKV (n=73)^+^** | **BKN_Low_ (n=25)** | **BKN_Hi/B+_ (n=7)** | **p value** |
| --- | --- | --- | --- | --- |
| HLA-A |  |  |  | 0.086 |
| 0 | 22/73 (30.1%) | 4/25 (16.0%) | 4/7 (57.1%) |  |
| ≥1 mismatch | 51/73 (69.9%) | 21/25 (84.0%) | 3/7 (42.9%) |  |
| HLA-B |  |  |  | 0.816 |
| 0 | 22/73 (30.1%) | 7/25 (28.0%) | 1/7 (14.3%) |  |
| ≥1 mismatch | 51/73 (69.9%) | 18/25 (72.0%) | 6/7 (85.7%) |  |
| HLA-DR |  |  |  | 0.800 |
| 0 | 36/73 (49.3%) | 14/25 (56.0%) | 3/7 (42.9%) |  |
| ≥1 mismatch | 37/73 (50.7%) | 11/25 (44.0%) | 4/7 (57.1%) |  |

^+^Data not available for one patient.

**Supplementary Table S9** Immunosuppression medication changes due to BKPyV viraemia

**Any level of BKPyV viraemia (BKV)**

| **At time of BKV diagnosis** | | | **Medication change due to BKV** | | |
| --- | --- | --- | --- | --- | --- |
| Immunosuppression | Number of episodes | Proportion of total episodes (%) | Proportion of BK viraemia episodes with change of medication due to BKV (%) | Change | Number of episodes with medication change |
| Everolimus, tac, pred | 1 | 1/41 (2.4%) | 1/1 (100%) | Reduce dose of pred | 1 |
| Pred, aza, tac | 6 | 6/41 (14.6%) | 3/6 (50%) | Stop aza | 3 |
| Pred, tac, MMF | 10 | 10/41 (24.4%) | 5/10 (50%) | Reduce dose of MMF | 2 |
|  |  |  |  | Reduce dose of pred and tac | 1 |
|  |  |  |  | Stop pred | 1 |
|  |  |  |  | Reduce dose of tac | 1 |
| Sirolimus, aza, pred | 1 | 1/41 (2.4%) | 0/1 (0%) |  |  |
| Tac, MMF | 22 | 22/41 (53.7%) | 10/22 (45.5%) | Reduce MMF | 3 |
|  |  |  |  | Reduce tac | 1 |
|  |  |  |  | Reduce tac and MMF | 3 |
|  |  |  |  | Reduce tac, replace MMF with pred | 1 |
|  |  |  |  | Replace MMF with pred | 1 |
|  |  |  |  | Stop MMF | 1 |
| Tac, MMF at diagnosis with pred added during BK viraemia due to concurrent acute rejection | 1 | 1/41 (2.4%) | 1/1 (100%) | Reduce pred | 1 |

**BKN_Low_**

| **At time of BKV diagnosis** | |  | **Medication change due to BKV** | | |
| --- | --- | --- | --- | --- | --- |
| Immunosuppression | Number of episodes | Proportion of low level BKV episodes (%) | Proportion of BKV episodes with change of medication due to BKV (%) | Change | Number of episodes with medication change |
| Pred, aza, tac | 5 | 5/34 (14.7%) | 2/5 (40%) | Stop aza | 2 |
| Pred, tac, mmf | 9 | 9/34 (26.5%) | 4/9 (44.4%) | Reduce pred and tac | 1 |
|  |  |  |  | Stop pred | 1 |
|  |  |  |  | Reduce MMF | 1 |
|  |  |  |  | Reduce tac | 1 |
| Sirolimus, aza, pred | 1 | 1/34 (2.9%) | 0/1 (0%) |  |  |
| Tac, MMF | 18 | 18/34 (52.9%) | 6/18 (29.4%) | Reduce MMF | 2 |
|  |  |  |  | Reduce tac | 1 |
|  |  |  |  | Stop MMF | 1 |
|  |  |  |  | Reduce tac and MMF | 2 |
| Tac, MMF at diagnosis with pred added during BK viraemia due to concurrent acute rejection | 1 | 1/34 (2.9%) | 1/1 (100%) | Reduce pred | 1 |

**BKN_Hi/B+_**

| **At time of BKV diagnosis** | |  | **Medication change due to BKV** | | |
| --- | --- | --- | --- | --- | --- |
| Immunosuppression | Number of episodes | Proportion of high level BKV episodes | Proportion of BKV episodes with change of medication due to BKV (%) | Change | Number of episodes with medication change |
| Everolimus, tac, pred | 1 | 1/7 (14,3%) | 1/1 (100%) | Reduce pred | 1 |
| Pred, aza, tac | 1 | 1/7 (14.3%) | 1/1 (100%) | Stop aza | 1 |
| Pred, tac, mmf | 1 | 1/7 (14.3%) | 1/1 (100%) | Reduce MMF | 1 |
| Tac, MMF | 4 | 4/7 (57.1%) | 4/4 (100%) | Reduce MMF | 1 |
|  |  |  |  | Reduce tac, replace MMF with pred | 1 |
|  |  |  |  | Reduce tac and MMF | 1 |
|  |  |  |  | Replace MMF with pred | 1 |

Pred= prednisolone, Tac= tacrolimus, MMF= mycophenolate mofetil, BKV= Bk viraemia

**Supplementary Figure S1** Kidney function at time of diagnosis of BKPyV viraemia compared to the average reading during periods of viraemia.


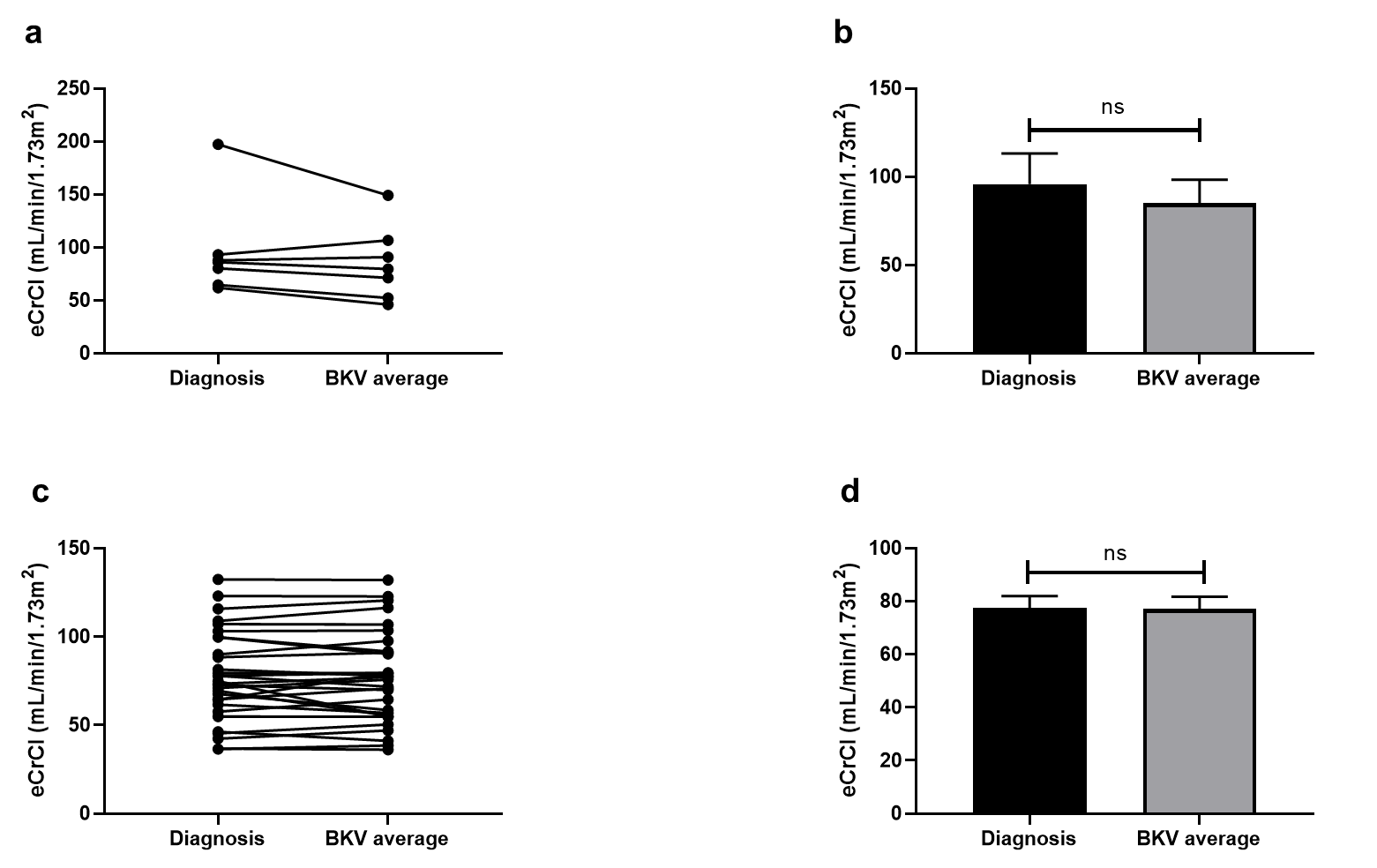


To understand how kidney function changed during periods of viraemia, eCrCl values for each patient were calculated at time of viraemia onset (‘diagnosis’) and compared to the mean eCrCl recorded during viraemia for each patient (all serum creatinine values available during periods of viraemia were included in the analysis). **a, b** BKN_Hi/B+_ patients (ns p= 0.710, Mann–Whitney U test). **c, d** BKN_Low_ patients (ns p= 0.956, Mann–Whitney U test). Bar charts show mean eCrCl and error bars represent standard error of the mean.
